# Glycans Instructing Immunity: The Emerging Role of Altered Glycosylation in Clinical Immunology

**DOI:** 10.3389/fped.2015.00054

**Published:** 2015-06-11

**Authors:** Jonathan J. Lyons, Joshua D. Milner, Sergio D. Rosenzweig

**Affiliations:** ^1^Genetics and Pathogenesis of Allergy Section, Laboratory of Allergic Diseases, National Institute of Allergy and Infectious Diseases, National Institutes of Health, Bethesda, MD, USA; ^2^Immunology Service, Department of Laboratory Medicine, Clinical Center, National Institutes of Health, Bethesda, MD, USA; ^3^Primary Immunodeficiency Clinic, National Institute of Allergy and Infectious Diseases, National Institutes of Health, Bethesda, MD, USA

**Keywords:** glycosylation, congenital disorders of glycosylation, immunodeficiency, allergy, infection susceptibility

## Abstract

Protein glycosylation is an important epigenetic modifying process affecting expression, localization, and function of numerous proteins required for normal immune function. Recessive germline mutations in genes responsible for protein glycosylation processes result in congenital disorders of glycosylation and can have profound immunologic consequences. Genetic mutations in immune signaling pathways that affect glycosylation sites have also been shown to cause disease. Sugar supplementation and *in vivo* alteration of glycans by medication holds therapeutic promise for some of these disorders. Further understanding of how changes in glycosylation alter immunity may provide novel treatment approaches for allergic disease, immune dysregulation, and immunodeficiency in the future.

## Introduction

Glycans are biologically important sugars, which may be single monosaccharides or oligosaccharides present as branched structures. Most commonly, glycans are linked covalently via glycosidic linkages to either nitrogen provided by an asparagine residue (N-linked glycans) or by oxygen from serine or threonine residues (O-linked glycans). Glycosylation of the human proteome is estimated to be at least 40% and glycans can comprise up to 90% of the total molecular weight of certain glycoproteins (1, 2). At least 2% of the genome is dedicated to creating, curating, maintaining, and recognizing glycans, and the loss of any component of these processes can result in dire consequences or incompatibility with life (3). To date, over 100 recessive Mendelian disorders resulting from hypomorphic mutations in glycosylation pathways have been identified, which result in the group of diseases known as congenital disorders of glycosylation (CDGs) (4). It is important to note that while approximately 2% of the genome encodes the requisite proteins needed for normal glycosylation, glycan structures themselves are not encoded. Therefore, glycans are an important form of post-translational modification and an epigenetic mechanism that can regulate gene expression.

Derangements in glycosylation patterns can occur in multiple ways and contribute to the complex clinical phenotypes seen in CDGs. Specific glycosylation patterns are required for the normal activity of many immune molecules. Changes in patterns of glycosylation may lead to impaired protein expression, altered protein ligand function, and significant alterations in immune pathway signaling. The impact of glycans on specific immune molecules has been studied extensively in model systems (5, 6). However, the global effect of defective glycosylation on immune function is less well characterized. This review will explore glycan biology in the context of immune function, describe known immunodeficiency syndromes resulting from disordered glycosylation, and discuss the potential for clinical manipulation of glycosylation patterns in patients using enzymatic inhibitors and sugar supplementation.

## Glycans

It is useful to group glycans into basic categories, which generally compartmentalize with localization and function. While additional glycoforms and important exceptions exist to these generalizations, a basic understanding of the roles of specific linkages and glycans will serve to instruct the remainder of this review.

### N-linked

**Figure 1:**
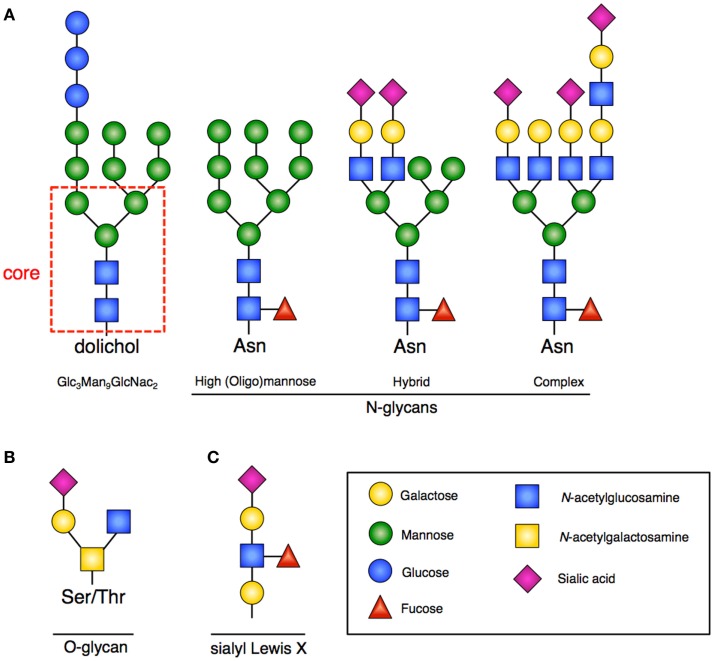
**Glycan structures**. **(A)** Representative examples of *N*-glycans increasing in complexity from left to right. Far left is the 14-sugar precursor (Glc_3_Man_9_GlcNac_2_) with conserved core (indicated in red dashed box). This structure is transferred *en bloc* from dolichol to asparagine (Asn) to form other *N*-glycans. Increasing branching and complexity is enabled by addition of GlcNAc to the core structure. **(B)** Representative example of an *O*-glycan structure; no conserved core exists. **(C)** Sialyl-Lewis-X (sLex) structure that would be frequently found at the terminus of poly-*N*-acetyllactosamines.

N-linked glycosylation is the most common form of covalent protein modification in human cells and is commonly found on secretory and membrane-bound glycoproteins. N-linked glycans are so-named because the sugars are covalently linked to nitrogen groups on asparagine residues of proteins when present in a consensus amino acid sequence (Asn-X-Ser/Thr). There are three general classes of *N*-glycans: oligomannose (high-mannose) type, complex type, and hybrid type. All share a common pentasaccharide (five sugar) core. The oligomannose type only has mannose residues attached to the core, the complex type has only *N*-acetylglucosamine (GlcNAc), which results in branching from the core, and the hybrid type contains both mannose and GlcNAc (2) (Figure 1A).

The process of creating N-linked glycans begins on the cytoplasmic surface of the endoplasmic reticulum (ER) where GlcNAc is attached to the lipid carrier molecule dolichol. Following completion, the glyan core is transferred to the ER lumen where the requisite 14-sugar oligosaccharide precursor (Glc_3_Man_9_GlcNac_2_) is completed (Figure 1A). This structure is then transferred *en bloc* to a nascent peptide chain containing the consensus sequence. Subsequent glycan remodeling begins in the ER: glucose residues are removed, as are a variable number of the mannose residues. Removal of these residues occurs during the protein folding cycle, which is guided by a host of molecular chaperones (7). *N*-glycans facilitate proper protein folding by enhancing polypeptide solubility and by recruiting the lectin chaperones calnexin and calreticulin. Misfolded proteins are recognized by their expression of a specific *N*-glycan (terminal α1,6-linked mannose) structure contributing to ER stress and the unfolded protein response (UPR) (8). When a disordered polypeptide segment is identified, the terminally misfolded glycoprotein is retro-translocated to the cytoplasm where it is targeted for ubiquitination and proteasomal degradation, a process called endoplasmic-reticulum-associated protein degradation (ERAD) (9). If folding properly occurs, the glycoprotein is transited to the Golgi where additional mannose residues may be removed and multiple additional sugars (GlcNAc, galactose, fucose, sialic acid) and other moieties (sulfate, phosphate) may be added. The complete glycoproteins are then trafficked to their ultimate destinations.

### O-linked

In contrast to N-linked glycans, O-linked glycans are formed once the protein has transited to the Golgi, and occur by step-wise addition of monosaccharides. Formation occurs by the covalent addition of primarily *N*-acetylgalactosamine (GalNAc) to the oxygen group present on serine (Ser) or threonine (Thr) residues (Figure 1B). A variety of different core structures for *O*-glycans have been described based upon initial and subsequent sugar addition. Many large intracellular glycoproteins contain O-linked glycans, but membrane-bound and secreted proteins may also contain these residues (2).

### *O*-GlcNAc modification

Within the cytoplasm and nuclear compartments, a distinct form of glycosylation called O-GlcNAcylation may also occur. It is distinguished by the nature and cellular location of sugar addition. The amino-sugar uridine diphosphate *N*-acetylglucosamine (UDP-GlcNAc) provides the substrate for O-linked addition of single GlcNAc molecules at Ser and Thr residues (10). Addition of GlcNAc in this context functions much like a phosphate group; it is transient, may be added and removed multiple times, and generally exists only as a single sugar. Similar to the way in which protein phosphorylation may affect protein activation, *O*-GlcNAc addition may compete with phosphorylation, and following addition to acceptor sites by *O*-GlcNAc transferase (OGT) can alter protein activity without affecting subcellular localization or phosphorylation state (11).

### Sialyl-lewis-X

A common outer structural element on both *N*- and *O*-glycans is the sub-terminal disaccharide *N*-acetyllactosamine (LacNAc). In the Golgi, these disaccharides can be added at multiple sites of glycans resulting in poly-*N*-acetyllactosamines (frequently called polylactosamine). The outer LacNAc residues are frequently capped by sialic acid residues. Subsequent fucosylation gives rise to the sialyl-Lewis-X (sLex) motif (Figure 1C), which facilitates cell–cell interactions by providing an important ligand for Selectins, mediating processes such as intercellular adhesion (12).

## Congenital Disorders of Glycosylation

Numerous biologic functions are dependent on glycans and a complete loss of function in any step required for glycan formation is frequently incompatible with life, though in some cases compensatory mechanisms exist (4). Recessive hypomorphic mutations in any step may likewise result in a CDG. Genotype–phenotype correlations in these disorders are often inconsistent and difficult to establish. In part, because of the variability and complex nature of the phenotypes seen in CDGs, this group of disorders has only been recognized clinically in the past 30 years.

Currently, there are over 100 known CDGs affecting virtually every step of glycan synthesis, transfer, and curation and they have been assembled into two basic groups. By historical convention, type I defects result from impaired glycan formation. Patients with type I disorders are characterized by the absence of *N*-glycans (specifically sialic acid residues) at consensus sequences. These may be detected clinically by mass spectroscopic evaluation of glycan patterns on serum transferrin, which contains two such consensus sequences. Type II disorders result from a failure to trim or appropriately curate glycans once they have been added to target proteins or lipids. When evaluating serum proteins in these disorders, the majority of N-glycosylation sites are occupied, but glycan complexity and diversity are altered (13).

## Glycosylation is Critical to Normal Immune Function

In addition to mechanisms broadly important to immunity such as cell–cell adhesion and signal transduction, several specific innate and adaptive immunologic pathways have demonstrated a dependence on appropriate glycosylation.

### Innate immunity

Glycoproteins are critical for normal cell–cell interactions. This is perhaps most apparent in leukocyte trafficking (14). In the context of an inflammatory response, the first step of mobilizing cells to sites of inflammation involves rolling on the vascular endothelium. This process is mediated by the glycan ligand sLex and the C-type lectin receptors Selectins (12). Absence of sLex, as seen in patients with *SLC35C1* mutations, results in impairment of this process and leukocyte adhesion deficiency type 2 (discussed separately) (15). In addition to sLex, other glycoprotein ligands for the Selectins are important to leukocyte homing and trafficking exist and include GlycCAM1, P-selectin glycoprotein ligand 1 (PSGL1), and α_M_β_2_ integrin (16).

Toll-like receptors, cytokines, and cytokine receptors also comprise glycoproteins and have multiple N-glycosylation sites (17, 18). Experimentally disrupting glycosylation sites on these glycoproteins has demonstrated that expression and/or function is reduced or abolished *in vitro* when glycan expression is altered. Both loss of glycans at predicted sites and gain of novel glycan consensus sites have been shown to result in altered signaling (19, 20).

In a study examining all reported variants in the Human Gene Mutation Database, it was found that approximately 1% of mutations present created new *N*-glycan consensus sequences. The potential impact of this finding was demonstrated among patients with mutations in Mendelian susceptibility to mycobacterial disease (MSMD) caused by interferon-gamma receptor 2 (IFN-γR2) insufficiency (21). A protein positive *IFNGR2* mutation identified in three patients with MSMD resulted in the addition of a glycosylation site by creating an additional *N*-glycan consensus sequence (T168N). Other mutations in *IFNGR2* have also been reported to result in disordered N-glycosylation through unclear mechanisms (R114C, S124F, G141R, and G227R). Remarkably in all cases, *in vitro* cellular responses to IFN-γ were improved by exogenous *N*-glycan modification. Similar mutations resulting in *N*-glycan perturbations in type I cytokine receptors have also been reported. Such mutations in granulocyte colony-stimulating factor (G-CSF) receptor and interleukin-21 receptor (IL-21R) have been shown to cause severe congenital neutropenia (SCN) and a combined immunodeficiency syndrome, respectively (22, 23).

### Adaptive immunity

Glycans appear to dictate multiple aspects of adaptive immunity affecting both T cells and B cells. Beginning early in T cell ontogeny, glycans appear to regulate fate decisions and diversity (24–27). The C-type lectin galectin-1 expressed in the thymus modulates T cell receptor (TCR) signal strength and contributes to negative selection (28). Antigen receptors on T cells (TCR) and B cells (BCR), as well as major histocompatibility complex (MHC) molecules are likewise glycoproteins (26). In mouse models employing tetramers, alteration in specific glycan motifs has been shown to have significant effects in skewing TCR repertoires (29). The T cell co-receptors CD4 and CD8, and several other molecules critical in dictating T cell fates including CTLA-4 and Notch receptors, are glycoproteins and their expression and function are dependent upon normal glycosylation. Understanding the specific contribution that altered glycosylation may have on T cell differentiation *in vivo* is an active area of investigation.

In addition to the effect glycans have on antigen receptor signaling, immunoglobulin activity following class-switch recombination is also a glycan dependent process. Immunoglobulin G (IgG), IgE, and IgA all contain glycosylation sites that dictate receptor binding thus affecting trafficking and function (30). Alterations of glycan composition have been shown to dramatically alter IgG half-life *in vitro* and *in vivo* (31, 32). In humanized animal models, alternative glycosylation motifs in the Fc groove of IgG also result in non-canonical signaling and have implications in IgG effector function (33).

## Immune Consequences of Altered Glycosylation

**Figure 2:**
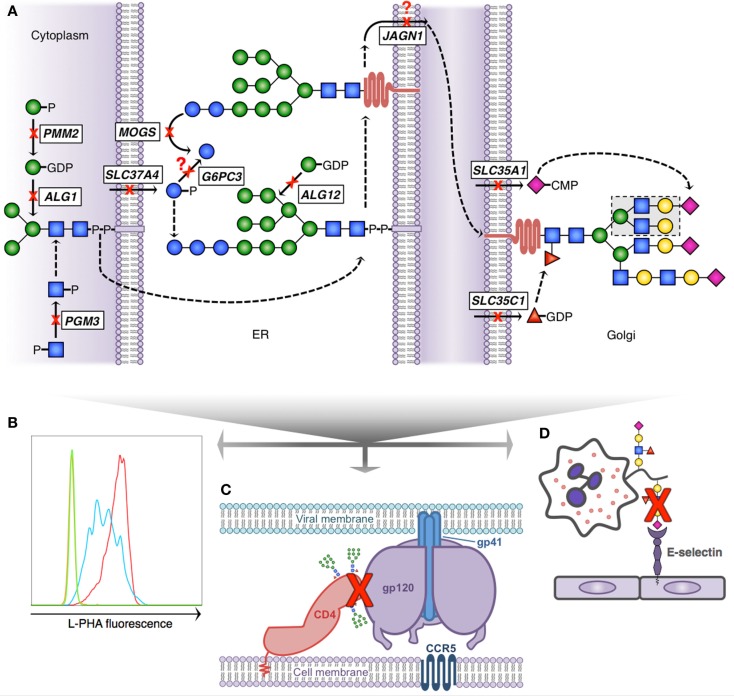
**Glycosylation pathway defects with known immunologic consequences**. **(A)** Simplified scheme demonstrating N-linked glycosylation and 10 known gene defects resulting in altered glycosylation and immunologic disease; dashed arrows indicate presence of non-depicted enzymatic steps; question marks depict theoretical blocks for which the mechanistic defect remains speculative. *N*-glycan formation begins in the cytoplasm (left) where phosphoglucomutase 3 (*PGM3*) and phosphomannomutase 2 (*PMM2*) provide key sugar substrate and beta-1,4-mannosyltransferase (*ALG1*) adds the first mannose. The core glycan is transferred to the ER lumen where alpha-1,6-mannosyltransferase (*ALG12*) adds an additional mannose and glucose-6-phosphate transporter 1 (*SLC37A4*) and glucose-6-phosphatase-β (*G6PC3*) potentially affect availability of glucose to complete the 14-sugar precursor (Glc_3_Man_9_ GlcNac_2_). Following transfer of the growing glycan to a nascent protein, mannosyl-oligosaccharide glucosidase (*MOGS*) participates in glycan remodeling prior to transit of the glycoprotein to the Golgi, a process facilitated in part by jagunal homolog1 (*JAGN1*). In the Golgi CMP-sialic acid transporter (*SLC35A1*) and GDP-fucose transporter 1 (*SLC35C1*) provide additional sugar substrate to complete complex *N*-glycan formation. **(B)** Reduced branching *N*-glycan pattern seen in patients with *PGM3* deficiency (blue) detected using a fluorescently labeled lectin (L-PHA) to quantitate expression on lymphocytes by flow cytometry (green, unstained; red, control). **(C)** Schematic depiction of HIV infection initiation requiring the interaction of several glycoproteins (CD4, gp120 trimer, and gp41 trimer). Reduced complexity of N-glycans seen in *MOGS* deficiency, as depicted by the presence of high-mannose residues in this figure, results in impaired viral entry, and reduced infectivity of newly formed virions *in vitro*. **(D)** Depiction of impaired leukocyte trafficking due to reduced sialyl-Lewis-X (sLex) binding to E-selectin. *SLC35C1* mutations result in leukocyte adhesion deficiency, type 2 (LAD2), and mutations in *SLC35A1* result in impaired sLex expression on neutrophils (indicated by red X). In LAD2, this results in a leukocyte trafficking defect in patients and impaired immunity.

Given that expression and function of numerous critical immune molecules are dependent on normal glycosylation; one might predict that there would be multiple primary immunodeficiency diseases (PIDDs) caused by defective glycosylation. While work to extensively immunophenotype CDG patients continues, we will discuss several PIDDs already identified resulting from glycosylation defects (Table 1; Figure 2).

**Table 1:** **Inherited glycosylation defects resulting in immune dysfunction**.

Gene	Protein	Glycosylation defect	Immune defect
*G6PC3*	Glucose-6-phosphatase-β	*N*-glycan and *O*-glycan complexity	Neutropenia
*SLC37A4*	Glucose-6-phosphate transporter 1	*N*-glycan and *O*-glycan complexity	Neutropenia
*PGM3*	Phosphoglucomutase 3	*N*-glycan and *O*-glycan expression	Combined immunodeficiency; neutropenia; atopy
*PMM2*	Phosphomannomutase 2	*N*-glycan expression	Neutrophil chemotaxis; humoral responses
*ALG1*	Beta-1,4-mannosyltransferase	*N*-glycan expression	Humoral immunodeficiency
*ALG12*	Alpha-1,6-mannosyltransferase	*N*-glycan expression	Humoral immunodeficiency
*MOGS*	Mannosyl-oligosaccharide glucosidase	*N*-glycan complexity	Low immunoglobulins; reduced susceptibility to *N*-glycosylated enveloped viruses
*SLC35A1*	CMP-sialic acid transporter	Global sialylation	Neutropenia
		O-mannosylation^a^	
*SLC35C1*	GDP-fucose transporter 1	Global fucosylation	Leukocyte trafficking
*JAGN1*	Jagunal homolog1	*N*-glycan fucosylation	Neutropenia

*^a^O-mannosylation defect demonstrated on α-dystroglycan *in vitro**.

### SLC37A4 and G6PC3 deficiencies

Glucose-6-phosphatase, catalytic, 3 (*G6PC3*) encodes the catalytic subunit of glucose-6-phosphatase-β (G6Pase), a ubiquitously expressed ER-resident protein. This enzyme is critical for liberating free glucose, catalyzing the final step of gluconeogenesis and glycogenolysis (34). G6Pase activity is dependent upon substrate – G6P within the ER – supplied by the glucose-6-phosphate transporter 1 (G6PT1) (35). G6PT1 is encoded by solute carrier family 37, member 4 (*SLC37A4*). Autosomal recessive mutations in *G6PC3* and *SLC37A4* both result in similar forms of SCN characterized by susceptibility to severe bacterial and fungal infections. At least seven different homozygous damaging mutations in *G6PC3* among Turkish, Greek, Arab, Persian, and German kindreds have been identified (36–40). Intermittent thrombocytopenia, cardiac defects, prominent superficial veins, microcephaly, and sensorineuronal hearing loss have also been associated with these mutations (41). In humans, *G6PC3*-deficient neutrophils appear dysfunctional and demonstrate evidence of ER stress, with enhanced propensity toward apoptosis (42). In addition to SCN, mutations in *SLC37A4* cause glycogen storage disease (type Ib) characterized by hypoglycemia, hepatomegaly, hyperlipidemia, growth failure, hyperuricemia, inflammatory bowel disease, and lactic acidosis (43). Loss of either G6PC3 or SLC37A4 results in reduced glycosylation of the NADPH oxidase system, as well as a reduction in *N*- and *O*-glycan complexity characterized by truncation of complex branching sugars (44). Whether this is due to lack of substrate or metabolic failure remains to be determined.

### PGM3 deficiency

Recently, autosomal recessive hypomorphic mutations in phosphoglucomutase-3 (*PGM3*) were identified in nine kindreds by three independent groups (45–47). The clinical phenotype described was quite broad potentially representing the degree of impairment in enzymatic function. Absent *Pgm3* is embryonically lethal in mice and predicted to be incompatible with life in humans (48). Hypomorphic *Pgm3* function in mice results in a bone marrow failure phenotype and a similar phenotype was observed among three patients harboring deleterious *PGM3* mutations, who presented early in life with T^−^B^−^NK^+^ severe combined immunodeficiency (SCID). Two patients underwent hematopoietic stem cell transplant (HSCT) – one received 6/6 HLA-matched cord blood at 4 months of age, the other underwent HLA-identical sibling-mobilized peripheral blood stem cell transplantation at 6 years of age. Both had successful engraftment and the patients were healthy 14 and 6 months out from transplant at the time of publication. In addition to a SCID phenotype, compound heterozygous and homozygous hypomorphic mutations were identified among six families presenting with a hyper-IgE phenotype, characterized by moderate to severe atopic dermatitis, significant elevations in IgE and connective tissue abnormalities; dysmorphic appearance and neurocognitive impairment were also common to all three patient cohorts. Unique to *PGM3*-deficient patients with hyper-IgE phenotypes was an increase in Th17 cells and autoimmunity.

PGM3 normally interconverts GlcNAc-1-phosphate and GlcNAc-6-phosphate. The fundamental defect in *PGM3* deficiency appears to be just downstream with a failure in normal UDP-GlcNAc formation. UDP-GlcNAc is a critical nucleotide-sugar that provides the GlcNAc substrate for *N*- and O-linked glycosylation as well as *O*-GlcNAc modification, thus making this a type I CDG. In the absence of adequate GlcNAc, a failure to form the core *N*-glycan would be predicted, and indeed reduced *N*-glycans were demonstrated in these patients. Further work is underway to better characterize defects in *O*-glycans and *O*-GlcNAc modification, and to understand the specific pathways being affected, which lead to such profound immunophenotypes.

### PMM2 deficiency

In the steps following GlcNAc addition during core *N*-glycan formation, mannose (Man) residues are added from UDP-Man in a manner similar to UDP-GlcNAc. *PMM2* encodes phosphomannomutase-2, an enzyme analogous to PGM3, which interconverts Man-6-phosphate to Man-1-phosphate. Failure in this step due to autosomal recessive mutations in *PMM2* results in insufficient mannose substrate and CDG-Ia, the most common CDG, also known as Jaeken syndrome (49). Presentation may be quite variable. Some patients present in infancy with a severe multi-system syndrome comprised of neurologic impairment, GI disease, and failure to thrive, while other patients survive well into adulthood but demonstrate intellectual disability, associated with neurologic, musculoskeletal, and endocrine defects. Of those patients presenting early in childhood, a high mortality is observed in the first year of life, and infection is the most common cause. Because of this finding, a systematic immune evaluation of a cohort of CDG-Ia patients was undertaken which revealed impairment in neutrophil chemotaxis and poor vaccine responses among affected individuals (50). Clinical improvement as measured by a reduced incidence of infection was observed when patients were placed on intravenous immunoglobulin (IVIG) replacement.

### ALG1 and ALG12 deficiencies

Just as insufficient substrate for mannosylation caused by *PMM2* mutations results in immunologic sequelae, impaired enzymatic activity limiting mannosylation directly, results in a similar clinical phenotype. Asparagine-linked glycosylation 1 homolog (*ALG1*) encodes beta-1,4-mannosyltransferase, the enzyme responsible for addition of the first mannose residue to the growing core *N*-glycan (Figure 1A). Autosomal recessive loss-of-function mutations in *ALG1* result in CDG-Ik, characterized by dysmorphic features, neurologic impairment, and humoral deficiency characterized by decreased B cells and undetectable IgG in the context of severe protein loss (due to nephrotic syndrome in one patient, and GI losses in another) (51–54). Asparagine-linked glycosylation 12 homolog (*ALG12*) encodes alpha-1,6-mannosyltransferase, the enzyme responsible for adding the eighth mannose residue to the core *N*-glycan. Damaging recessive mutations in *ALG12* result in CDG-Ig, characterized by dysmorphia, psychomotor retardation, failure-to-thrive, male genital hypoplasia, low IgG, and humoral deficiency complicated by recurrent bacterial infections (55).

### MOGS deficiency

Within the ER after GlcNAc and Man residues are added to the core *N*-glycan, glucose is added to cap and complete its production. The next step in glycan processing involves removal of these terminal glucose residues by mannosyl-oligosaccharide glucosidase (MOGS). Failure to remove terminal glucose residues is seen in patients with autosomal recessive damaging mutations in *MOGS*, resulting in CDG-IIb (56). Clinically, the patients display developmental, neurologic, and musculoskeletal defects. The enzymatic defect leads to accumulation of high-mannose-type *N*-glycans and a failure to form complex-type *N*-glycans (normal structures shown in Figure 1A) with profound immunologic outcomes (31). Patients exhibit hypogammaglobulinemia due to a significantly reduced IgG half-life. Despite these impairments, patients do not have an increase in clinical infections, and are paradoxically protected from *N*-glycosylated enveloped virus infections as a consequence of abnormal glycoprotein expression and formation.

### SLC35A1 deficiency

The common outer structural element of sLex is created by fucosylation of sialic acid capped poly-*N*-acetyllactosamines (as described above and shown in Figure 1C). Also, as previously discussed, Selectins recognize this motif and bind to it facilitating the first step in leukocyte trafficking. For sialylation to occur, the sugar nucleotide precursor cytosine-5-monophospho (CMP)-sialic acid must be transported into the ER. Solute carrier family 35, member 1 (*SLC35A1*) encodes the CMP-sialic acid transporter that accomplishes this. CDG-IIf is caused by autosomal recessive loss-of-function mutations in *SLC35A1* (57). To date, one patient has been reported with damaging heterozygous mutations in *SLC35A1* presenting with a syndrome characterized by neutropenia, macrothrombocytopenia, recurrent invasive bacterial infection and hemorrhage. Complete absence of sLex was reported on patient neutrophils as well; however, *in vitro* trafficking and responses appeared unaffected. The patient underwent bone marrow transplantation at 34 months, and died from refractory respiratory failure at 37 months following a complicated course, which included graft-versus-host disease and pulmonary hemorrhage.

### SLC35C1 deficiency (leukocyte adhesion deficiency, type 2)

As mentioned, fucosylation of sialic acid residues is required for sLex formation. The GDP-fucose transporter, which provides substrate for this addition (also called fucosyltransferase I), is encoded by *SLC35C1*. The transporter is located on the membrane of the Golgi where it normally functions to move cytosolic GDP-fucose into the Golgi lumen to act as a substrate for fucosylation of developing glycans. Loss of this transporter due to autosomal recessive mutations results in leukocyte adhesion deficiency, type 2 (LAD2), also known as CDG-IIc. These patients demonstrate a failure to appropriately fucosylate glycans and cannot make sLex. Patient leukocytes are unable to appropriately reach sites of infection, resulting in recurrent severe bacterial infections with the hallmark of neutrophilia and little or absent purulence at the infected site (15). As with other CDGs the clinical phenotype is variable and complex, but can include neurologic and developmental deficits, as well as dwarfism. Fucosylation also plays an important role in ABO blood group determinant and patients with this syndrome exhibit the Bombay blood group type.

### JAGN1 deficiency

Impaired fucosylation has also been identified among individuals with autosomal recessive loss-of-function mutations in jagunal homolog 1 (*JAGN1*). Fourteen patients from nine families have been identified with homozygous *JAGN1* mutations, resulting in SCN (type 6) (58, 59). Both maturational arrest of myeloid precursors in the bone marrow and enhanced apoptosis were seen in patient neutrophils. *JAGN1* encodes a ubiquitously expressed protein that localizes to the ER and participates in protein trafficking. Complete knockout of *Jagn1* is embryonically lethal in mice. For unclear reasons, disruption of this protein results in abnormal N-glycosylation. Defective fucosylation on branching *N*-glycans was identified both in *JAGN1*-deficient human neutrophils and in murine neutrophils carrying a hematopoietic lineage-specific deletion of *Jagn1*. However, other blood cells appear to be grossly unimpaired.

## Therapeutic Manipulation of Glycans

Because glycosylation is a post-translational modification, it is conceivable that some CDGs and immune disorders would be responsive to treatments that can alter glycans *in vivo*. There has been interest in treating CDG patients with excess or alternative sugar substrate in order to drive or shunt defective pathways and promote production of the deficient sugar product. While not all genetic deficiencies previously targeted are associated with identified immune defects, the approaches have been instructive and thus are discussed. Medications that alter glycan expression in patients with intact glycosylation pathways have also been investigated for use in the treatment of viral infections, and are briefly discussed.

### Fucose supplementation in LAD2

Among LAD2 patients with impaired GDP-fucose transport to the site of sugar addition in the Golgi, oral fucose supplementation has demonstrated mixed efficacy (60–63). In one cohort, a patient was administered oral fucose at 25 mg/kg, and these doses were slowly escalated to a maximum of 492 mg/kg over approximately 9 months of continuous supplementation. Therapy resulted in normalization of neutrophil counts and sLex, a reduction of incident infection, and improvement in physical and psychomotor growth and development (60). In a second cohort, two patients were given a loading dose of 2.5–5 g, followed by daily supplementation with 200 mg/kg. After 1 month, the dose was increased to 1 g/day in five divided doses and continued for 1 year without significant improvement in fucosylated protein expression or other clinical parameters (61). In the latter study, dosing may have been insufficient to achieve normalization. A fourth patient was treated with oral fucose at 165 mg/kg, increased to 1000 mg/kg divided in five daily doses over approximately 9 months. Neutrophil number and glycan fucosylation were rescued with a reduction in incident infection. Unfortunately, the success of therapy was tempered by induction of autoimmune neutropenia with coincident expression of intact H-antigen on erythrocytes in this individual (63).

### Mannose supplementation in MPI and PMM2 deficiencies

Mannosphosphate isomerase (*MPI*) interconverts fructose-6-phosophate, derived from glucose, and mannose-6-phosphate providing additional substrate for phosphomannomutase-2 (*PMM2*). Autosomal recessive defects in *MPI*, like *PMM2* discussed previously, deplete the pool of GDP-mannose available for glycosylation. *MPI* deficiency presents in childhood with protein losing enteropathy, coagulopathy, fibrotic liver disease, and failure to thrive. In contrast to *PMM2* deficiency, neurologic impairment does not appear to be a feature. A patient with *MPI* deficiency was treated with 100 mg/kg three times daily, increased after 8 months to 150 mg/kg five times daily (64). Normalization of serum transferrin glycan expression, resolution of GI bleeding, and resolution of protein losing enteropathy with normalization of total serum protein was observed. A 5-year follow-up on this patient demonstrated persistent efficacy of oral mannose replacement (65). By striking contrast, patients with *PMM2* deficiency, treated for 9 days with 100 mg/kg every 3 h, demonstrated no clinical or laboratory improvement on enteral mannose therapy (66).

### Galactose supplementation in PGM1 deficiency

More recently, galactose supplementation has been used in one cohort of six patients from five kindreds with phosphoglucomutase 1 (*PGM1*) deficiency (67). *PGM1* normally interconverts glucose-1-phosphate (Glc-1-P) and glucose-6-phosphate (Glc-6-P). Glc-1-P can also be enzymatically derived from galactose-1-phosphate and dietary supplementation with galactose may provide excess substrate to overcome reduced Glc-1-P resulting from *PGM1* deficiency. As with other CDGs, the clinical phenotype of patients with *PGM1* deficiency is variable and complex, including musculoskeletal abnormalities, growth and developmental delay, and endocrine abnormalities. Supplementation with 0.5–1.0 mg/kg/day of galactose resulted in normalization of *N*-glycan expression on serum transferrin and other serum proteins. Clinical improvement was also reported in two patients characterized by partial resolution of hypogonadotropic hypogonadism, cessation of rhabdomyolysis episodes, and no progression of hepatopathy or cardiac disease.

### GlcNAc and triacetyluridine supplementation in PGM3 deficiency

Among patients with *PGM3* deficiency exogenous GlcNAc *in vitro* has been demonstrated to restore UDP-GlcNAc levels in patient fibroblasts, and to partially rescue cellular abnormalities (45). A clinical protocol to provide *PGM3*-deficient patients with supplemental GlcNAc and triacetyluridine will likely begin enrollment in early Summer 2015.

### MOGS inhibitors and viral immunity

Mannosyl-oligosaccharide glucosidase inhibitors are one example of how manipulation of glycans may provide therapeutic benefits in the general population. Whether through co-opting lectin receptors, such as DC-SIGN, or by canonical protein receptor–ligand interactions, glycoproteins of enveloped viruses mediate docking and entry of particles into host cells (68, 69). In both lectin and protein-based glycoprotein recognition, the glycan structure is critical to successful infection, as demonstrated in patients with CDG-*MOGS* (30). Several MOGS inhibitors have been developed, and have demonstrated the ability to reduce viral entry, replication, and virulence *in vitro* (70–72). In a clinical trial, patients treated with the MOGS inhibitor miglustat in combination with zidovudine had lower plasma viral loads and higher CD4^+^ T cell counts compared to those treated with zidovudine alone, without significant side effects (73). Other drugs that alter glycosylation, such as kifunensine and nelfinavir, have also displayed therapeutic promise (20, 74). In the future, additional drugs, which alter glycosylation, may provide useful primary or adjunctive treatments, or regimens for prophylaxis, particularly in the context of enveloped viral infections.

### Limitations of supplementation and additional clinical approaches

Therapeutic sugar supplementation, while a promising clinical intervention, remains untested in many CDGs. Because any one CDG is rare, and each enzymatic deficiency results in a discrete defect, caution must be taken in generalizing success from one CDG to another, even within the same pathway (e.g., *PMM2* and *MPI*). Furthermore, excess sugar substrate from supplementation may lead to alternate biosynthetic pathway activation. This in turn may potentially lead to toxic metabolite formation, induction of autoimmunity, or loss of partial viral protection. Because of these complex issues, no consensus recommendations exist to guide therapy at this time.

While no medications currently exist to specifically target improvement of defective enzymatic activity, enzyme replacement therapy, and gene therapy are under investigation to treat these disorders, and have shown promise in some immunodeficiency diseases. HSCT has been shown to be effective in treating SCID caused by *PGM3* deficiency. How transplantation in this compartment may improve disease manifestations in other organ systems (e.g., neurologic function) remains to be seen.

## Conclusion

Altered glycosylation is a well-established feature in many disease states. Particular glycan patterns are known to be associated with oncogenesis and tumor progression, as well as a host of illnesses and pathologic processes occurring later in life, including multiple sclerosis, Alzheimer’s disease, paroxysmal nocturnal hemoglobinuria, rheumatoid arthritis, IgA nephropathy, and polycystic liver disease (75–81). CDGs, the archetypal inherited disorders of altered glycosylation likewise produce myriad clinical features, and may result in profound immunologic defects and immunodeficiency. Discrete alterations in glycan expression due to mutations in immune molecules (e.g., *IFNGR2*) have also been shown to result in primary immunodeficiency. Understanding the mechanisms by which altered glycans resulting from germline genetic changes exert their dominant effects on immunologic function may provide novel approaches for targeting immune defects in the future. Furthermore, manipulation of glycans by medication or dietary supplementation may prove beneficial not only to CDG patients but also to PIDD patients who share common immune phenotypes.

## Conflict of Interest Statement

The authors declare that the research was conducted in the absence of any commercial or financial relationships that could be construed as a potential conflict of interest.
